# Bioefficacy of long-lasting insecticidal nets against pyrethroid-resistant populations of *Anopheles gambiae s.s.* from different malaria transmission zones in Uganda

**DOI:** 10.1186/1756-3305-6-130

**Published:** 2013-05-02

**Authors:** Michael Okia, Richard Ndyomugyenyi, James Kirunda, Anatol Byaruhanga, Seraphine Adibaku, Denis K Lwamafa, Fred Kironde

**Affiliations:** 1 National Malaria Control Programme, Ministry of Health, P.O. Box 7272, Kampala, Uganda; 2 C/O Vector Control Division, Ministry of Health, P.O. Box 7272, Kampala, Uganda; 3 School of Entomology C/OVector Control Division, Ministry of Health, P.O. Box 7272, Kampala, Uganda; 4 Ministry of Health, P.O. Box 7272, Kampala, Uganda; 5 Department of Biochemistry, College of Health Sciences, Makerere University, P.O. Box 1661, Kampala, Uganda

**Keywords:** Long-lasting insecticidal nets (LLIN), Pyrethroid-resistant *An.gambiae* s.s, Uganda

## Abstract

**Background:**

There are major concerns over sustaining the efficacy of current malaria vector control interventions given the rapid spread of resistance, particularly to pyrethroids. This study assessed the bioefficacy of five WHO-recommended long-lasting insecticidal nets (LLINs) against pyrethroid-resistant *Anopheles gambiae* field populations from Uganda.

**Methods:**

Adult *An. gambiae* from Lira, Tororo, Wakiso and Kanungu districts were exposed to permethrin (0.75%) or deltamethrin (0.05%) in standard WHO susceptibility tests. Cone bioassays were used to measure the bioefficacy of four mono-treated LLINs (Olyset®, Interceptor®, Netprotect^®^ and PermaNet^®^ 2.0) and one combination LLIN (PermaNet^®^ 3.0) against the four mosquito populations. Wireball assays were similarly conducted to determine knockdown rates. Species composition and *kdr* mutation frequency were determined for a sample of mosquitoes from each population. Chemical assays confirmed that test nets fell within target dose ranges.

**Results:**

*Anopheles gambiae s.s.* predominated at all four sites (86 - 99% of *Anopheles spp*.) with moderate *kdr* L1014S allelic frequency (0.34 – 0.37). Confirmed or possible resistance to both permethrin and deltamethrin was identified for all four test populations. Reduced susceptibility to standard LLINs was observed for all four populations, with mortality rates as low as 45.8% even though the nets were unused. The combination LLIN PermaNet^®^3.0 showed the highest overall bioefficacy against all four *An. gambiae s.l.* populations (98.5 - 100% mortality). Wireball assays provided a more sensitive indicator of comparative bioefficacy, and PermaNet 3.0 was again associated with the highest bioefficacy against all four populations (76.5 – 91.7% mortality after 30 mins).

**Conclusions:**

The bioefficacy of mono-treated LLINs against pyrethroid-resistant field populations of *An. gambiae* varied by LLIN type and mosquito population, indicating that certain LLINs may be more suitable than others at particular sites. In contrast, the combination LLIN PermaNet 3.0 performed optimally against the four *An. gambiae* populations tested. The observed reduced susceptibility of malaria vectors to mono-treated LLINs is of particular concern, especially considering all nets were unused. With ongoing scale-up of insecticidal tools in the advent of increasing resistance, it is essential that those interventions with proven enhanced efficacy are given preference particularly in areas with high resistance.

## Background

Malaria remains a major public health problem, causing an estimated 225 million disease cases and 781,000 deaths per year, especially among children aged less than five years [1]. The disease is transmitted by anopheline mosquitoes and vector control is one of the most important means of malaria prevention. There is evidence that the use of insecticide-treated nets (ITNs) on a large scale decreases malaria related morbidity and mortality [2,3] and for this reason, the use of ITNs has been considered an important tool in the Roll Back Malaria (RBM) strategy.

Unlike conventional ITNs which lose effective insecticide after one or two washes and maintain bioefficacy for a maximum of 6–12 months, long-lasting insecticidal nets (LLINs) in which insecticide is either incorporated into the fibre during extrusion or coated on the fibre following extrusion, retain effectiveness against susceptible *Anopheles* spp. vectors for up to 20 standard WHO laboratory washes and 3 years of recommended usage under field conditions [4]. All LLINs are currently treated with pyrethroids due to their relative safety for humans at low dosage, repellent properties, rapid knock-down rates and killing effects [5]. However, pyrethroid resistance in mosquito vectors as reported in many African countries [6] could limit the efficacy of LLINs as shown by findings of decreased efficacy of LLINs in Benin, Mali and Zanzibar [7-9].

Insecticide resistance is mediated either by mutations in the target site of the insecticide or its active metabolites (target site resistance), through enzymatic modification of insecticides to produce non-toxic metabolites (metabolic detoxification), via behaviour resistance or through reduced penetration of the insecticide into the vector species [10]. Several factors can select for resistance in mosquito vector species, such as overuse of insecticide, whether in ITNs, indoor residual spraying (IRS) or through agricultural applications which account for huge insecticide inputs of almost all available classes of insecticides [11,12]. In Uganda, there is widespread insecticide resistance in the main malaria vector species, *An. gambiae s.s, An. arabiensis* and *An. funestus*[13-18]. This resistance is due to both target site (*kdr*) and metabolic mechanisms and there is cross-resistance between DDT and pyrethroids. There are currently no reports of organophosphate resistance but resistance to carbamates including propoxur has been reported [13-18].

The current strategy of the National Malaria Control Programme (NMCP) in Uganda is based on effective case management and vector control using LLINs and IRS. Insecticide resistance monitoring is therefore essential to guide implementation of more effective and sustainable vector control. There have been limited data on comparative efficacy of World Health Organization (WHO)-recommended LLINs against field-derived populations of *Anopheles* spp. from different transmission zones within single countries. Rather, efficacy has largely been measured in specific areas via experimental huts with only one or two nets assessed in relation to controls. In Uganda, one study showed progressive reductions over a 10 year period in susceptibility of *An. funestus* from the western region to nets treated with three different insecticides. However, non-standard bioassay techniques were used and mosquitoes from five parishes were pooled for assessments [15]. In the absence of experimental huts and in order to assess susceptibility to LLINs of multiple local malaria vector species, the Uganda NMCP initiated the present study using WHO-recommended LLINs against local *Anopheles gambiae* populations. Outcomes are expected to be applied in evidence-based decision making on the most appropriate LLINs for application in malaria prevention and control in specific regions of the country.

## Methods

### Mosquito collections

**Figure 1:**
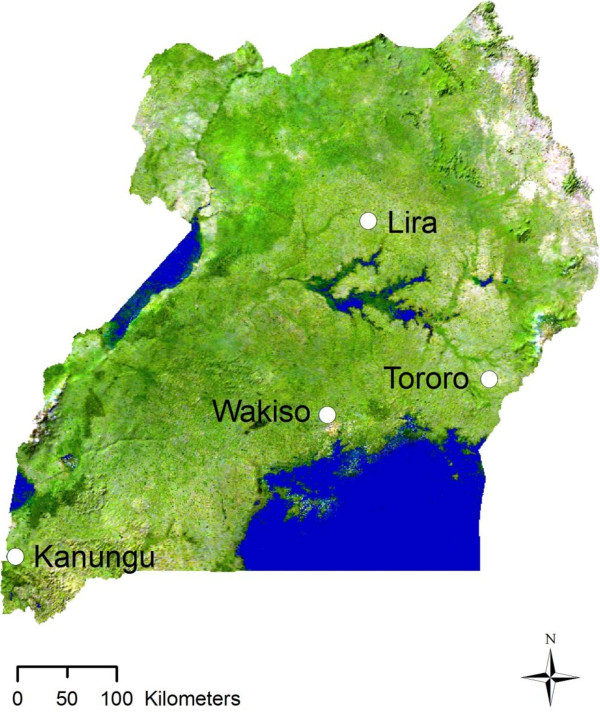
**Map of Uganda showing the origin of the four *An. gambiae s.l. *populations used in WHO susceptibility tests, species and resistance mechanisms investigations and LLIN bioassays.**

Collections were conducted in April and October 2011 in the districts of Lira, Tororo, Wakiso and Kanungu located in Northern, Eastern, Central and Western regions of Uganda, respectively (Figure 1). In these four districts, malaria transmission levels range from very high (Lira, Tororo) to medium-high (Wakiso) to low (Kanungu) (Additional file 1). Previous studies identified the presence of *kdr* mutations in *An. gambiae s.l.* from Tororo district in the eastern part of the country, Apac district in northern Uganda near the current study district of Lira, in the central part of the country and in Kyenjojo and Kanungu in the western part of the country, with *kdr* frequencies ranging from 25% to 30% in these districts [14,16-19]. Metabolic resistance mechanisms have also been implicated in populations from Tororo with a significant increase in esterase activity detected [17].

Female *Anopheles* spp. adult mosquitoes were collected via standard resting catches from houses and larvae were collected from breeding sites within the study districts, and all were transported to the Vector Control Division (VCD) insectary in Kampala. Blood-fed and gravid females were allowed to oviposit, eggs were hatched and larvae were pooled for each collection district. Field-collected larvae were also pooled by collection district. All preimaginal stages were reared to adults under conditions of ambient temperature and humidity with 12:12 hours of light: dark cycle. Unfed adult females at 2 – 5 days post-emergence were identified morphologically as previously described [20] at the Centre for Research on Infectious Diseases Laboratory, College of Health Science Makerere University, Kampala. Only *An. gambiae s.l.* mosquitoes were used in WHO susceptibility tests and cone bioassays.

### Insecticide susceptibility and molecular testing

Standard WHO susceptibility tests were conducted to determine mortality rates (MT) following one hour of exposure to papers treated with either permethrin (0.75%) or deltamethrin (0.05%). Concurrent negative controls were run using untreated papers. Test populations were classified as susceptible (≥98% MT), possibly resistant (80-97%) or confirmed resistant (<80% MT) [21]. Mosquitoes used in controls were stored in contact with silica gel desiccant. A random sample of 100 mosquitoes from each site was used in species identification by restriction fragment length polymerase chain reaction [22] and determination of *kdr* mutation frequency by allele-specific polymerase chain reaction [23,24]. All PCR runs for *kdr* analyses included controls of wild type homozygote, heterozygote and *kdr* homozygote mosquitoes for both L1014S and L1014F.

### LLIN samples

All LLINs as well as the untreated control nets were obtained from the local market. All had an unknown storage history but were within the specified product shelf-life. LLINs included in the study were: Olyset^®^ Net (polyethylene with permethrin incorporated at 20 g/kg ± 3 g/kg), Interceptor^®^ (polyester coated with alphacypermethrin at 200 mg/m^2^ ± 25%), NetProtect^®^ (polyethylene with deltamethrin incorporated at 1.8 g/kg ± 25%), PermaNet^®^ 2.0 (polyester coated with deltamethrin at 55 mg/m^2^ ± 25%) and PermaNet^®^ 3.0 (polyethylene roof with deltamethrin incorporated at 2.8 g/kg ± 25% and piperonyl butoxide (PBO) incorporated at 4.0 g/kg ± 25% in the roof and sides coated with deltamethrin at 2.8 g/kg ± 25%). PBO is a synergist that increases the rate of penetration of insecticide into the insect [25] and inhibits the metabolic enzymes the mosquito uses to sequester or break-down the insecticide [26].

All LLINs were rectangular with sub-samples of 30 × 30 cm taken from the roof section (2 per net) and side sections (1 each from the upper and lower part of the two long sides of each net) to give a total of 6 sub-samples for each net for use in bioassays. Four nets of each type were used for a total of 24 sub-samples of each net type assessed via cone bioassays. Identical sub-sampling was performed in adjacent areas for reference samples used in chemical assays. All samples were rolled up and placed individually in a labelled clean aluminium foil prior to assays.

### LLIN chemical analyses

Assays were conducted via high performance liquid chromatography (HPLC) to confirm whether chemical concentrations were within product specifications for each individual LLIN. Analyses were conducted at an ISO IEC 17025-accredited laboratory. Deltamethrin was assessed by normal-phase HPLC according to CIP333/LN. Alpha-cypermethrin was extracted with n-hexane and 1, 4-dioxane (95:5 v/v) with the mixture then shaken and sonicated and filtered on a 0.45 mm teflon membrane, whereas for permethrin and PBO, hot xylene extraction was followed by drying, reconstitution and filtration after which both were assessed via HPLC.

### LLIN bioassays

Standard WHO cone bioassays [4] were used to determine bioefficacy of LLINs against field-derived populations as well as against a susceptible laboratory-reared *Anopheles gambiae s.s.* strain (Kisumu). The Kisumu colony was established at the Vector Control Division (VCD) of the Ministry of Health in Kampala in 2011, with full susceptibility (100% mortality) to permethrin (0.75%) and deltamethrin (0.05%) confirmed via standard WHO susceptibility tests prior to assays. At the VCD insectary, five non-blood fed 2-to 5-day old *Anopheles* females were exposed to each sub-sample for 3 minutes, removed and kept in holding containers with access to sugar solution. Knockdown (KD) was recorded at 60 minutes post-exposure and mortality (MT) was recorded after 24 hours. Two cone tests were conducted per sub-sample and per mosquito population including for the laboratory susceptible population such that 240 mosquito of each of the five populations were tested for each net type. Mosquitoes exposed to untreated nets were used as controls with all concurrent results discarded if MT was ≥20% and Abbott’s adjustment applied if MT was >5% for the controls.

Wireball assays were used to measure knockdown following 30 and 60 mins of continuous exposure to an LLIN in a wireball. This approach was included as it is of use where mortality rates may be lower and hence longer exposure times are required, or where high repellency of the insecticide may compel mosquitoes to rest on the cone interior rather than on the LLIN. Net sub-samples were wrapped around a wire frame of three intersecting circles of 15 cm in diameter with the netting secured around the frame in such a way that a “sleeve” was left through which 11 mosquitoes were introduced. Numbers of mosquitoes knocked down after 30 mins (KD_30_) and 60 mins (KD_60_) were recorded. Mosquitoes were then transferred to holding cups for 24-hour post-exposure readings. For each individual sub-sample, four wire-ball tests were conducted such that 44 mosquitoes were tested per sub-sample. With 3 sub-samples of each individual net and 3 nets of each type, a total of 396 mosquitoes were tested for each net type. Controls were run concurrently with interpretation as for cone bioassays.

### Data analyses

For cone bioassays, KD and MT were compared for individual samples via regression analyses. Data, aggregated for mosquito population, net type and net section, were assessed via ANOVA with Duncan’s multiple comparison procedure. Data were then combined for net sections and assays were repeated. Wireball assay data for KD_30_ and KD_60_ were similarly analyzed.

## Results

### Population characterisation and insecticide susceptibility

For the population analyses, the majority of collected females were morphologically identified as *An. gambiae s.l.* (391/400) with a small proportion identified as *An. funestus s.l.* (9/400). Molecular analyses indicated that at all four sites, *An. gambiae s.s.* predominated (Table 1). The *kdr* mutation L1014S was detected in 257 of the 363 *An. gambiae s.s.* successfully tested, with overall 29.2% homozygous wild type (SS), 70.5% heterozygous (RS) and one single homozygous resistant (RR) mosquito detected from Kanungu. The *kdr* allelic frequency was moderate at all sites, and varied from 0.34 at Lira to 0.37 at Wakiso. Genotype frequencies for all populations did not adhere to Hardy-Weinberg expectations. All *An. arabiensis* tested (14) were wild type. No L1014F mutations were observed in any species.

**Table 1:** **Species composition of *Anopheles spp. *and *kdr *mutation frequency in *An. gambiae s.s.*from the four study sites**

**Study site**	**Species**				***An. gambiae s.s.kdr*mutation**	
	**Identified (no.)**	***An. funestus *(%)**	***An. arabiensis *(%)**	***An. gambiae s.s. *(%)**	**Genotyped (no.)**	**L1014S frequency (%)**
Kanungu	98	2.0	0.0	98.0	94	36.7
Lira	100	0.0	1.0	99.0	97	33.5
Tororo	99	1.0	13.1	85.9	79	35.4
Wakiso	100	6.0	0.0	94.0	93	36.6

Only *An. gambiae s.l.* were used in further assays. WHO susceptibility tests confirmed resistance to both permethrin and deltamethrin for the populations from Lira and Tororo (Table 2). There was confirmed resistance to permethrin and possible resistance to deltamethrin for the population from Kanungu, and possible resistance to both pyrethroids for the population from Wakiso. At all the four sites, higher resistance to permethrin was identified than to deltamethrin at the standard tested dosages. The Kisumu laboratory strain of *An. gambiae s.s.* was 100% susceptible to both pyrethroids.

**Table 2:** **Susceptibility to permethrin and deltamethrin of *An. gambiae *adult female mosquitoes collected from four sites in Uganda and the laboratory *An. gambiae s.s. *(Kisumu) strain determined via standard WHO susceptibility tests**

	**Permethrin (0.75%)**			**Deltamethrin (0.05%)**		
**Mosquito population**	**Number exposed**	**24 h mortality (%)**	**Susceptibility status**	**Number exposed**	**24 h mortality (%)**	**Susceptibility status**^
Kanungu	100	68	Confirmed resistant	100	97	Possibly resistant
Lira	100	60	Confirmed resistant	100	71	Confirmed resistant
Tororo	100	53	Confirmed resistant	100	66	Confirmed resistant
Wakiso	100	90	Possibly resistant	100	94	Possibly resistant
Kisumu	100	100	Susceptible	100	100	Susceptible

^ Susceptible (≥98%), possibly resistant (80–97), confirmed resistant (<80%).

### LLINs and bioassays

All LLIN sub-samples had optimal bioefficacy (100% KD and 100% MT), against the susceptible *An. gambiae s.s.* (Kisumu), strain in cone bioassays, with the exception of Interceptor (78.8% and 80.0% KD for upper and lower sides respectively, and 97.5% MT for upper side) and Olyset (92.5% KD for upper side). For wireball assays with the susceptible strain, KD_30_ ranged between 84.8 and 93.3% with 100% MT at 24 hours post-exposure for all LLINs. Chemical analyses confirmed that all LLINs exceeded the specified lower cut-off level for insecticide (or synergist) concentration though there were two instances where LLIN sub-samples slightly exceeded the upper limits i.e., roof of PermaNet 2.0 and sides of Interceptor (Table 3).

**Table 3:** Target concentration and range and mean insecticidal or synergist concentration measured via high performance liquid chromatography for roof and side sub-samples of five different LLIN types used in bioefficacy evaluations

		**Target concentration**			**Mean measured concentration**	
**Net type**	**Chemical**	**Units**	**Mean**	**Range**	**Roof**	**Sides**
PermaNet 3.0	Deltamethrin	g/kg	2.8 (sides)	2.1 - 3.5	-	3.1
		g/kg	4 (roof)	3.0 - 5.0	3.9	-
	Piperonyl butoxide	g/kg	25 (roof)	18.8 - 31.3	18.7	-
PermaNet 2.0	Deltamethrin	mg/m^2^	55	41.3 - 68.8	69.4	65.6
NetProtect	Deltamethrin	g/kg	1.8	1.4 - 2.3	1.6	1.6
Interceptor	Alpha-cypermethrin	mg/m^2^	200	150.0 - 250.0	171.0	251.0
Olyset	Permethrin	g/kg	20	17.0 - 23.0	21.0	21.6

An overall association was identified between KD and MT for cone bioassays on individual sub-samples (n = 720; R^2^ = 0.8903; P <0.0001), while associations on aggregated data showed correlation between KD and MT for Interceptor against all four mosquito populations, Olyset for 3 populations, and for the remaining LLINs two populations only (P < 0.05 for all specified). Comparisons of the bioefficacy of net sections (roof, upper sides, lower sides) indicated no difference among net types with the exception of Olyset against both the Kanungu and Tororo strains for both KD and MT (P < 0.05). Thus, there was no significant difference observed in the deltamethrin plus PBO roof and the deltamethrin-only sides of PermaNet 3.0, presumably because bioefficacy of the three sections was high against all four populations (≥87.9% KD and ≥ 97.5% MT). As such, data were aggregated by net type for subsequent analyses with data presented for MT.

**Figure 2:**
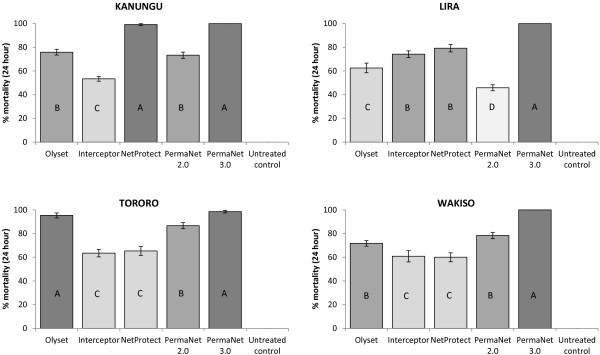
**Bioefficacy (mean % 24-hour mortality) in cone bioassays of WHO-recommended LLINs against field-derived *An. gambiae *populations from four sites in Uganda.** Vertical lines indicate standard error (n = 24). The same letter and shading within graphs indicates no significant difference (via Duncan’s multiple comparison procedure at P < 0.05).

Reduced susceptibility to LLINs was observed for all four field populations of *An. gambiae*. Bioefficacy varied between LLINs in cone bioassays with each of the four populations for both KD (P < 0.001 for all) and MT (P < 0.001 for all). Mean MT differed by 46.7% (range: 53.3-100%) for the Kanungu population, 54.2% (range: 45.8-100%) for the Lira population, 35.0% (range: 63.5-98.5%) for the Tororo population and 40.0% (range: 60.0-100) for the Wakiso population (Figure 2). PermaNet 3.0 exhibited the highest bioefficacy against all the four populations (98.5 – 100%). When data were analyzed via multiple comparison methods, PermaNet 3.0 performed significantly better than the mono-treated LLINs at Lira and Wakiso, and equal best with NetProtect at Kanungu and Olyset at Tororo. Each of the mono-treated LLINs also varied in bioefficacy for the four different populations for both KD (P < 0.001) and MT (P < 0.001). For PermaNet 3.0, there was no identifiable difference in bioefficacy against the four populations for either KD (P = 0.1011) or MT (P = 0.0890), presumably because bioefficacy was high against all populations. Conversely, there was also no significant difference in bioefficacy of the untreated net between populations since KD and MT were minimal for all.

Wireball assays also indicated differences in LLIN bioefficacy between net types for each of the four field populations, with PermaNet 3.0 resulting in the highest KD_30_ against all populations (76.5 – 91.7%) (Table 4). Bioefficacy also varied against the susceptible *An. gambiae s.s.* (Kisumu) strain (P < 0.001) and was highest for PermaNet 3.0 followed by PermaNet 2.0 and then the other LLINs, indicating that KD_30_ from wireball assays may be a more sensitive indicator of bioefficacy than KD and MT from cone bioassays. Bioefficacy of specific net types also varied against the different populations for the mono-treated LLINs (P < 0.001) and in contrast to the cone bioassay data, also varied across populations for PermaNet 3.0 (P = 0.0063) with the lowest KD_30_ (76.5%) observed against the Kanungu population. There was a significant overall association between KD_30_ and KD_60_ (n = 348; R^2^ = 0.8844; P <0.0001), and bioefficacy of the net sections differed only for PermaNet 3.0 against the Kanungu population (P = 0.0160) and Olyset against the Lira population (P = 0.0240).

**Table 4:** **Bioefficacy in wireball assays (mean % 30-minute knockdown) of WHO-recommended LLINs against field-derived *An. gambiae *populations from four sites in Uganda and a susceptible laboratory *An. gambiae s.s. *strain (Kisumu)**

	**Net type**						**P-value**
**Mosquito population**	**Olyset**	**Interceptor**	**NetProtect**	**PermaNet 2.0**	**PermaNet 3.0**	**Untreated control**	
Kanungu	42.4^C^	45.5^C^	47.7^C^	63.6^B^	76.5^A^	0.0^D^	<0.0001
Lira	67.4^C^	76.5^B,C^	83.3^A,B^	70.5^C^	86.4^A^	0.0^D^	<0.0001
Tororo	76.5^A,B^	76.5^A,B^	83.3^A^	70.5^B^	86.4^A^	nt	0.0241
Wakiso	75.8^B^	41.7^D^	65.9^C^	81.8^B^	91.7^A^	0.0^E^	<0.0001
Kisumu	84.9^B^	86.4^B^	84.9^B^	90.2^A,B^	93.3^A^	0.0^C^	<0.0001

Same letter in rows indicates no significant difference (via Duncan’s multiple comparison procedure at P < 0.05).

nt: not tested due to limited numbers of mosquitoes available.

**Figure 3:**
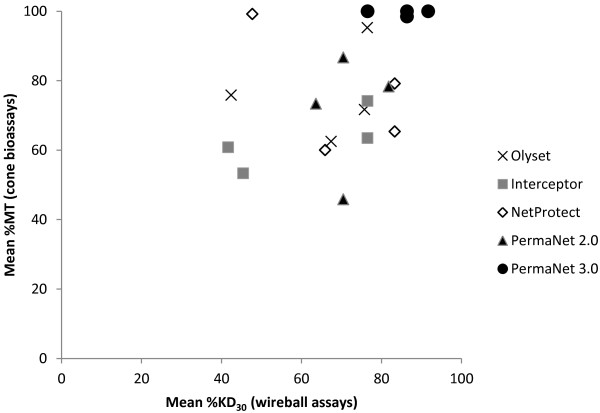
**Comparative bioefficacy of different LLIN types as determined via wireball versus cone bioassays for four populations of *An. gambiae *from Uganda.**

There was some concurrence between cone bioassays and wireball assays, especially for PermaNet 3.0, which exhibited high bioefficacy in both assay types (Figure 3). Considering both assay types, PermaNet 3.0 performed best or equal best against all four populations. NetProtect also performed well in cone bioassays against the Kanungu population and in wireball assays against the Lira population. Olyset performed well in cone bioassays against the Tororo population and three mono-treated LLINs also performed well in wireball assays against this population, indicating that the Tororo population was overall the most susceptible to LLINs. In general, cone bioassays indicated that LLINs had the lowest efficacy against the Lira and Wakiso populations (72.3 and 74.2% MT, respectively), but wireball assays indicated the lowest efficacy against the Kanungu population (55.2% KD_30_). Bioefficacy was highest against the Tororo population for both bioassays.

## Discussion

This is the first study to compare the response of field populations of malaria vectors from multiple sites in Uganda to WHOPES-recommended LLINs. Wide variations were observed in susceptibility to the different net types even within specific mosquito populations, with reduced susceptibility to pyrethroid-only LLINs observed for all four populations. Cone bioassays indicated that for two populations, a single mono-treated LLIN performed well (i.e., NetProtect at Kanungu and Olyset at Tororo), while optimal efficacy of the combination LLIN PermaNet 3.0 was observed for all four populations. Bioefficacy of LLINs differed by almost 50% against some populations (e.g. Kanungu had 53.3% MT with Interceptor and 100% MT with PermaNet 3.0 in cone bioassays).

Yewhalaw and colleagues [27] similarly observed reduction in the bioefficacy of standard LLINs against four pyrethroid-resistant *An. arabiensis* populations from the Jimma region of Ethiopia. In contrast to results herein, the PBO plus deltamethrin roof had higher bioefficacy than the pyrethroid-only sides of PermaNet 3.0. While the Ethiopia data provide evidence to indicate that PBO was effectively restoring susceptibility of the *An. arabiensis* populations to deltamethrin, this could not be demonstrated in the current investigation since bioefficacy of the deltamethrin-only sides of PermaNet 3.0 was optimal against the Uganda *An. gambiae* populations. Studies comparing the bioefficacy of PermaNet 3.0 versus deltamethrin- or permethrin-only LLINs in experimental huts have been conducted in numerous countries with results indicating that comparative bioefficacy will largely depend on the levels and types of resistance mechanisms present in the local vector species [28-32]. Data from some of these studies were applied in a malaria transmission model to compare PermaNet 3.0 to the deltamethrin-only PermaNet 2.0 under conditions of high net coverage (80%), with outputs indicating that PermaNet 3.0 (new and washed 20 times) had consistently higher impact on entomological inoculations rates across four sites with pyrethroid resistant *Anopheles spp.*[33].

Observed variation in susceptibility of *Anopheles* populations to pyrethroid-only LLINs indicates that particular LLINs may be more suitable for deployment in specific regions. This is due to anticipated differences in bioefficacy depending on characteristics of individual mosquito populations. This is currently seldom a consideration in selection of LLINs for wide-scale deployment, which is usually guided by availability, price and other factors such as user acceptability of the polymer type (e.g. polyester versus polyethylene). Reliance on phenotypic susceptibility status to select nets by active ingredient is also not appropriate since results from WHO susceptibility tests cannot be extrapolated to expected results from LLIN bioassays. In this study, susceptibility to the permethrin LLINs was highest for the population found to be least susceptible to permethrin (Tororo). Comparative bioefficacy evaluations using local vector populations such as presented in this study provide valuable data to inform selection of appropriate interventions. The consistently optimal bioefficacy of PermaNet 3.0 indicates that this combination LLIN represents a viable option for areas with pyrethroid-resistant *Anopheles spp*.

The current study provides compelling further evidence of increasing pyrethroid resistance in Uganda, which is consistent with observations from other studies [13-19]. The *kdr* mutation (L1014S) was detected at a moderate frequency (34–37%) in *An. gambiae s.s.* across all four sites. Although metabolic resistance assays were not conducted, it is likely that these *kdr* mutations may in part be contributing to the observed reductions in efficacy of the standard LLINs. In another study in selected areas in Uganda with resistant vector populations, *kdr* frequency was found to be notably higher in *P. falciparum*-infected mosquitoes, which contributed to 70% of the malaria transmission during the dry season [18]. Although fitness cost was not assessed, this potential for higher infectivity may have enormous implications for malaria transmission and might jeopardize current resistance management strategies. It also indicates that such resistance may be affecting the bioefficacy of insecticide-based vector control interventions, such as LLINs. This requires confirmation, using standard WHO approaches such as Phase II experimental hut trials or robust longitudinal and multi-site village trials since the observations in this study were based only on cone and wireball bioassays conducted under laboratory conditions. However, the low KD and MT rates observed give some indication that there may be reductions in the ability of mono-treated LLINs to kill mosquitoes under field conditions [34], and that their continued use may have limited impact on malaria prevention and control in Uganda. Reductions in efficacy of insecticidal interventions ,due to resistance, has been noted elsewhere, such as in South Africa, Benin, Mali and Equatorial Guinea [7,8,35,36]. Accordingly, further investigations in Uganda are warranted.

The reduced susceptibility to permethrin and deltamethrin observed for the four field populations of *An. gambiae s.l.* was similarly noted in assessments conducted between 2004 to 2006 and in 2009 and 2011 in Central and Eastern Uganda, with *kdr* identified as the main resistance mechanism and metabolic resistance also implicated for Tororo district [18]. In the current study, resistance was higher in Tororo, Kanungu and Lira than in Wakiso districts perhaps due to the historical widespread use of insecticides such as organochlorines and pyrethroids in the cotton growing districts of Tororo and Lira and in the tea cultivation fields of Kanungu. Resistance may also have arisen from selective pressure exerted due to the rapid scale-up of malaria interventions, such as LLINs and IRS. The fact that the *kdr* mutational assortments in the four tested populations did not meet Hardy-Weinberg expectations is a further indication that the populations are likely currently undergoing selective pressure. Interestingly, the frequency of L1014S in the *An. gambiae s.s.* from Tororo in this study (35%), was significantly lower than that reported in 2008 (86%), but was more similar to earlier reports from 2006 (47%) and 2002 (29%) [16,18]. While rapid geographical spread of insecticide resistance alleles has been noted from ongoing longitudinal studies [37], evidence of such rapid reversion to wild type is limited and thus this warrants further investigation. The presence of multiple resistance mechanisms in malaria vector species in Tororo may have severe implications for control efforts and further testing for metabolic resistance mechanisms in Uganda should be prioritized.

The WHO recommends that action against insecticide resistance should be immediate and pre-emptive, not responsive [37]. Data as presented here provides evidence for guiding decisions on the selection of LLINs with the highest efficacy for use in specific regions of Uganda. Evidence-based decision making was successfully applied by the Uganda National Malaria Control Programme in 2009 when there was a switch from the use of pyrethroids to carbamates for IRS, following results from resistance studies indicating reduced susceptibility to pyrethoids in major malaria vectors (Additional files 2 and 3) in Northern Uganda. More comprehensive studies will be needed to ascertain the bioefficacy of LLINs in Uganda, although the WHO cautions against awaiting indisputable proof of control failures before taking action against insecticide resistance [37]. With the new global initiative of the Roll Back Malaria (RBM) partnership to scale up for impact (SUFI), PermaNet 3.0 may be the most appropriate LLINs to use for malaria prevention particularly in the Northern and Central regions of Uganda where pyrethroid-resistance is already high and there is proof of increased bioefficacy relative to standard LLINs. Despite concerted efforts by the Ministry of Health to control malaria in Lira and neighbouring districts in the Northern region, malaria has remained a challenge. A survey conducted in the adjacent district of Apac in 2001–2002 found perennial holoendemic malaria with parasite prevalence rates of 70-90% in children less than 10 years of age [38]. In the subsequent 2009 survey conducted in Apac district, age sero-prevalence curves gave no indication of recent changes in malaria transmission intensity in the area [39]. This calls for urgent scale up of malaria prevention interventions with proven bioefficacy to rapidly achieve high coverage and resulting individual and community protection from malaria.

Current WHO guidelines recommend combining ITNs and IRS in various transmission settings, especially in areas with holoendemic and epidemic malaria [40]. LLINs and IRS could be used together in the same households in Northern and Eastern regions to suppress malaria transmission. However, if LLINs are to be combined with IRS for malaria prevention and control, the selection of appropriate LLIN types and IRS chemicals should be done with caution to avoid further exacerbating existing resistance. Products with the highest proven bioefficacy against local vector populations should be selected and IRS chemicals should differ from pyrethroids in their mode of action. In the absence of novel classes of insecticides, organophosphate- or carbamate-based IRS could be used where both LLIN and IRS are applied to form part of an insecticide resistance management strategy [41]. Encouragingly, recent insecticide susceptibility evaluations in Uganda found high susceptibility to carbamates and organophosphates in malaria vector populations (Additional file 2). A parasitemia survey in children conducted in late 2010 in three contiguous districts of Northern Uganda found that parasitemia levels were lower in two districts that had been sprayed with carbamates ( 37.0% and 16.7% positive smears) compared to a non-sprayed district ( 49.8% positive smears) [42]. There is a need for routine resistance surveillance and ongoing LLIN and IRS bioefficacy assessments against local vector populations so that products with significantly reduced efficacy relative to other available options can be replaced accordingly.

## Conclusions

Pyrethroid resistance in malaria vectors in Uganda is high and is likely to limit the impact of LLINs. Evaluation of the efficacy of various LLINs against *An. gambiae* populations from different malaria transmission zones has provided valuable information on wide variations depending on the population and LLIN being tested. Such information can be used to make rational decisions for selecting LLINs with the highest anticipated bioefficacy without waiting for indisputable proof of control failures from more comprehensive studies. Monitoring the efficacy of LLINs should be undertaken regularly in order to guide policy on selection and distribution of LLINs.

## Competing interests

The authors declare no competing interests.

## Authors’ contributions

MO, JK, AB, SA and JR conceived the study and designed the experiments. FK supervised the genotyping for species identification and *kdr* determinations. MO, JK AB, RN and FK analysed the data, drafted and wrote the manuscript. All authors have read and approved the final manuscript.

## Supplementary Material

Additional file 1 Map to show malaria endemicity by district and entomological inoculation rates for specific sites in Uganda. Click here for file

Additional file 2 **Susceptibility to selected insecticides of adult *An. gambiae s.l. *from various districts in Uganda between August and October 2009.** Click here for file

Additional file 3 **Susceptibility to selected insecticides of adult *An. gambiae s.l. *from various districts in Uganda between October and November 2011.** Click here for file

## References

[B1] WHO WHO World Malaria Report 2010 2010 Geneva: World Health Organization 136

[B2] D’ Alessandro U Olaleye BO Mcquire W Langerock P Bennett S Aikins MK Thomson MC Cham MK Cham BA Greenwood BM Mortality and morbidity from malaria in Gambian children after introduction of a treated bed nets programme Lancet 1995 345 479 483 10.1016/S0140-6736(95)90582-0 7861874

[B3] Lengeler C Insecticide-treated bed net and curtains for preventing malaria Cochrane Database Syst Rev 2004 2 CDOO363 10.1002/14651858.CD000363.pub2 15106149

[B4] WHO Guidelines for Laboratory and Field testing of Long-lasting Insecticidal Mosquito Nets 2005 Geneva: World Health Organization WHO/CDS/WHOPES/GDCPP/2005.11

[B5] Zaim M Aitio A Nalasima N Safety of pyrethroid-treated mosquito nets Med Vet Entomol 2000 14 1 5 10.1046/j.1365-2915.2000.00211.x 10759305

[B6] Ranson H N’Guessan R Lines J Moiroux N Nkuni Z Corbel V Pyrethroid resistance in African anopheline mosquitoes: what are the implications for malaria control? Trends Parasitol 2010 27 91 98 10.1016/j.pt.2010.08.004 20843745

[B7] N'Guessan R Corbel V Akogbéto M Rowland M Reduced efficacy of insecticide-treated nets and indoor residual spraying for malaria control in pyrethroid resistance area, Benin Emerg Infect Dis 2007 13 199 206 10.3201/eid1302.060631 17479880 PMC2725864

[B8] Fane M Cisse O Traore CS Sabatier P *Anopheles gambiae* resistance to pyrethroid-treated nets in cotton versus rice areas in Mali Acta Trop 2012 122 1 6 10.1016/j.actatropica.2011.11.013 22154879

[B9] Haji KA Khatib BO Smith S Ali AS Devine GJ Coetzee M Majambere S Challenge for malaria elimination in Zanzibar: pyrethroid resistance in malaria vectors and poor performance of long-lasting insecticide nets Parasit Vectors 2013 6 82 10.1186/1756-3305-6-82 23537463 PMC3639098

[B10] Najera JA Zaim M Malaria Vector Control: Decision Making Criteria and Procedures for Judicious Use of Insecticides 2005 Geneva: World Health Organization Pesticide Evaluation Scheme

[B11] Bonabana-Wabbi J Dissertation, Assessing Factors Affecting Adoption of Agricultural Technologies: The Case of Integrated Pest Management (IPM) in Kumi District, Eastern Uganda 2008 Department of Agricultural and Applied Economics, Virginia Tech

[B12] Bonabana-Wabbi J Taylor BD Health and Environmental Benefits of Reduced Pesticide Use in Uganda: An Experimental Economics Analysis 2008 Orlando, Florida: Paper presented at the Joint Annual Meeting of the American Agricultural Economics Association and the American Council on Consumer Interests http://ageconsearch.umn.edu/bitstream/6441/2/469143.pdf

[B13] WHO Atlas of Insecticide Resistance of Malaria Vectors in the WHO African Region 2005 World Health Organization African Network for Vector Resistance, Regional Office for Africa 27pp

[B14] Verhaeghen K Van Bortel W Roelants P Backeljau T Coosemans M Detection of the east and west African *kdr* mutation in *anopheles gambiae* and *anopheles funestus* from Uganda using a new assay based on FRET/melt curve analysis Malaria J 2006 5 16 10.1186/1475-2875-5-16 PMC1409791 16504072

[B15] Rubaihayo J Tukesiga E Abaasa A Reduced susceptibility to pyrethroid insecticide treated nets by the malaria vector Anopheles gambiae s.l. in western Uganda Malaria J 2008 7 92 10.1186/1475-2875-7-92 PMC2432068 18503715

[B16] Ramphul U Boase T Bass C Okedi LM Donnelly MJ Insecticide resistance and its association with target-site mutations in natural populations of *Anopheles gambiae* from eastern Uganda Trans R Soc Trop Med Hyg 2009 103 1121 1126 10.1016/j.trstmh.2009.02.014 19303125

[B17] Morgan JC Irving H Okedi LM Steven A Wondji CS Pyrethroid resistance in an *Anopheles funestus* population from Uganda PLoS One 2010 5 7 e11872 10.1371/journal.pone.0011872 20686697 PMC2912372

[B18] Verhaeghen K Bortel WV Roelants P Okello PE Talisuna A Spatio-temporal patterns in *kdr* frequency in permethrin and DDT resistant *Anopheles gambiae s.s.* from Uganda AmJTrop Med Hyg 2010 82 566 573 10.4269/ajtmh.2010.08-0668 PMC2844549 20348500

[B19] Santolamazza F Calzetta M Etang J Distribution of knock-down resistance mutations in *Anopheles gambiae* molecular forms in west and west-central Africa Malaria J 2008 7 74 10.1186/1475-2875-7-74 PMC2405802 18445265

[B20] Gillies M Coetzee M A supplementary to Anophelinae of Africa South of the Sahara 1998 Johannesburg: South African Institute for Medical Research

[B21] WHO Test Procedures for Insecticide Resistance Monitoring in Malaria Vectors, Bio-efficacy and Persistence of Insecticide on Treated Surfaces 1998 Geneva: World Health Organization WHO/CDS/CPC/MAL/98.12

[B22] Scott JA Brogdon WG Collins FH Identification of single specimens of the *Anopheles gambiae* complex by the polymerase chain reaction AmJTrop Med Hyg 1993 49 520 529 10.4269/ajtmh.1993.49.520 8214283

[B23] Martinez-Torres D Chandre F Williamson MS Darriet F Bergé JB Devonshire AL Guillet P Pasteur N Pauron D Molecular characterization of pyrethroid knockdown resistance (*kdr*) in the major malaria vector *Anopheles gambiae s.s* Insect MolBiol 1998 7 179 184 10.1046/j.1365-2583.1998.72062.x 9535162

[B24] Ranson H Jensen B Vulule JM Wang X Hemingway J Collins FH Identification of a point mutation in the voltage-gated sodium channel gene of Kenyan *Anopheles gambiae* associated with resistance to DDT and pyrethroids Insect MolBiol 2000 9 491 497 10.1046/j.1365-2583.2000.00209.x 11029667

[B25] Moores G Bingham G Use of ‘temporal synergism’ to overcome insecticide resistance Outlook Pest Manag 2005 16 10.1564/16feb03 17304634

[B26] Ahmad M Denholm I Bromilow RH Delayed cuticular penetration and enhanced metabolism of deltamethrin in pyrethoid-resistant strains of *Helicoverpaarmigera* from China and Pakistan Pest Manag Sci 2006 62 805 810 10.1002/ps.1225 16649192

[B27] Yewhalaw D Asale A Tushune K Getachew Y Duchateau L Speybroeck Bio-efficacy of selected long-lasting insecticidal nets against pyrethroid resistant *Anopheles arabiensis* from South-Western Ethiopia Parasit Vectors 2012 5 159 10.1186/1756-3305-5-159 22871143 PMC3485103

[B28] Van Bortel W Chinh VD Berkvens D Speybroeck N Trung HD Coosemans M Impact of insecticide-treated nets on wild pyrethroid resistant *Anopheles epiroticus* populations from southern Vietnam tested in experimental huts Malaria J 2009 8 248 10.1186/1475-2875-8-248 PMC2781025 19874581

[B29] Tungu P Magesa S Maxwell C Malima R Masue D Sudi W Myamba J Pigeon O Rowland M Evaluation of PermaNet 3.0 a deltamethrin-PBO combination net against Anopheles gambiae and pyrethroid resistant Culexquinquefasciatus mosquitoes: an experimental hut trial in Tanzania Malaria J 2012 9 21 10.1186/1475-2875-9-21 PMC2817703 20085631

[B30] Corbel V Chabi J Dabire RK Etang J Nwane P Pigeon O Akogbeto M Hougard JM Field efficacy of a new mosaic long-lasting mosquito net (PermaNet 3.0) against pyrethroid-resistant malaria vectors: a multi-centre study in Western and Central Africa Malaria J 2010 9 113 10.1186/1475-2875-9-113 PMC2877060 20423479

[B31] N’Guessan R Asidi A Boko P Odjo A Akogbeto M Pigeon O Rowland M An experimental hut evaluation of PermaNet^®^ 3.0, a deltamethrin-piperonyl butoxide combination net, against pyrethroid-resistant *Anopheles gambiae* and *Culexquinquefasciatus* mosquitoes in southern Benin Trans Roy Soc Trop Med Hyg 2012 104 758 765 20956008 10.1016/j.trstmh.2010.08.008

[B32] Koudou B Koffi AA Malone D Hemingway J Efficacy of PermaNet 2.0 and PermaNet 3.0 against insecticide-resistant Anopheles gambiae in experimental huts in Cote d’Ivoire Malaria J 2011 10 172 10.1186/1475-2875-10-172 PMC3141592 21699703

[B33] Killeen GF Okumu FO N’ Guessan R Coosemans M Adeogun A Awolola S Etang J Dabire RK The importance of considering community-level effects when selecting insecticidal malaria vector control products Parasit Vectors 2011 4 160 10.1186/1756-3305-4-160 21838903 PMC3189155

[B34] Takken W Do insecticide-treated bed nets have an effect on malaria vectors? Trop Med Int Health 2002 7 1022 1030 10.1046/j.1365-3156.2002.00983.x 12460393

[B35] Hargreaves K Koekemoer L Brooke D *Anopheles funestus* resistant to pyrethroid insecticides in South Africa Med Vet Entomol 2003 14 181 189 10872862 10.1046/j.1365-2915.2000.00234.x

[B36] Sharp BK Ridl FC Govender D Kuklinski J Kleinschmidt Malaria vector control by indoor residual insecticide spraying on the tropical island of Bioko, Equatorial Guinea Malaria J 2007 6 52 10.1186/1475-2875-6-52 PMC1868751 17474975

[B37] WHO The Technical Basis for Coordinated Action Against Insecticide Resistance:Preserving the Effectiveness of Modern Malaria Vector Control 2010 Geneva: World Health Organization Global Malaria Programme

[B38] Okello PE Bortelw V Byaruhanga AM Correwyn A Roelants P Talisuna A D’ Allessandro U Coosemans M Variation in malaria transmission intensity in seven sites throughout Uganda AmJTrop Med Hyg 2006 75 219 225 16896122

[B39] Proietti C Pettinato DD Kanoi BN Ntege E Crisanti A Riley EM Egwang TG Drakeley C Bousema T Continuing intense malaria transmission in Northern Uganda AmJTrop Med Hyg 2011 84 830 837 10.4269/ajtmh.2011.10-0498 PMC3083756 21540398

[B40] WHO World Malaria Report 2009 2009 Geneva: World Health Organization

[B41] Djenontin A Clabi J Baldet T Irish S Pennetier C Hougard JM Corbel V Akogbeto M Chandre F Managing insecticide resistance in malaria vectors by combining carbamate-treated plastic wall sheeting and pyrethroid-treated bed nets Malaria J 2009 8 233 10.1186/1475-2875-8-233 PMC2776024 19843332

[B42] Steinhardt LC Adoke Y Nasr S Wiegand RE Rubahika D Serwanga A Wanzira H Lavoy G Kamya M Dorsey G Filler S The effect of indoor residual spraying on malaria and anaemia in a high transmission area of Northern Uganda Am Trop Med Hyg 2013 88 855 861 10.4269/ajtmh.12-0747 PMC3752748 23458956

