# Supplementary material for: Bioefficacy of long-lasting insecticidal nets against pyrethroid-resistant populations of Anopheles gambiae s.s. from different malaria transmission zones in Uganda
Source: Parasit Vectors. 2013 May 2;6:130. doi: 10.1186/1756-3305-6-130 (PMC3656772; doi:10.1186/1756-3305-6-130)

**Additional File 1**: Map to show malaria endemicity by district (shaded regions) and entomological inoculation rates for specific sites (pointers) in Uganda. Transmission level categories are indicated by colour, with very high (red), medium-high (orange), low (yellow) and very low or no malaria (cream).


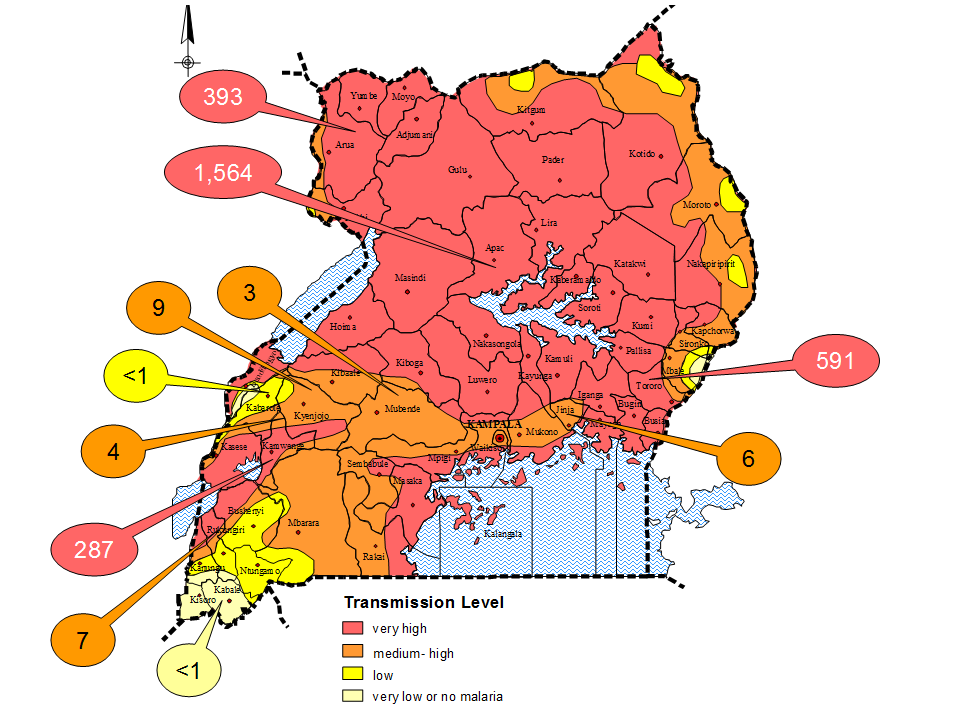

Supplement: Additional file 1 — Map to show malaria endemicity by district and entomological inoculation rates for specific sites in Uganda. [file 1756-3305-6-130-S1.docx]
