# Supplementary material for: Bioefficacy of long-lasting insecticidal nets against pyrethroid-resistant populations of Anopheles gambiae s.s. from different malaria transmission zones in Uganda
Source: Parasit Vectors. 2013 May 2;6:130. doi: 10.1186/1756-3305-6-130 (PMC3656772; doi:10.1186/1756-3305-6-130)
Supplement: Additional file 2 — Susceptibility to selected insecticides of adult An. gambiae s.l. from various districts in Uganda between August and October 2009. [file 1756-3305-6-130-S2.docx]

**Additional File 2**: 24-hour mortality (%) following exposure to selected insecticides in standard WHO susceptibility tests of adult *An. gambiae s.l*. from various districts in Uganda between August and October 2009. Test adults were non-blood fed and of known age (48-72 hours post-emergence), and were either the progeny of field-collected blood fed adult females or were reared from field-derived larvae.

| **Collection location** | **Wakiso** | | **Apac** | | **Kitgum** | | **Hoima** | | **Kanungu** | | **Tororo** | |
| --- | --- | --- | --- | --- | --- | --- | --- | --- | --- | --- | --- | --- |
| Origin of test adults | F1 of field adults | Field larvae | F1 of field adults | Field larvae | F1 of field adults | Field larvae | F1 of field adults | Field larvae | F1 of field adults | Field larvae | F1 of field adults | Field larvae |
| Vectors exposed (n) | 100 | 100 | 100 | 100 | 100 | 100 | 100 | 100 | 100 | 100 | 100 | 100 |
| DDT | 33 | 39 | 52 | 23 | 15 | 22 | 52 | 53 | 16 | 24 | 48 | 27 |
| Lambda-cyhalothrin | 95 | 86 | 68 | 49 | 27 | 31 | 89 | 80 | 27 | - | 75 | - |
| Etofenprox | - | 91 | 72 | - | - | 17 | 91 | - | 51 | - | 59 | - |
| Permethrin | - | 86 | 71 | - | - | 4 | 69 | - | 27 | - | 47 | - |
| Deltamethrin | - | 100 | 100 | 100 | 16 | 55 | 93 | - | 58 | - | 87 | - |
| Cyfluthrin | - | - | 100 | - | - | 14 | 88 | - | 88 | - | 93 | - |
| Pirimiphos-methyl | 100 | 100 | 100 | 100 | 100 | 100 | 100 | - | 100 | - | 100 | - |
| Malathion | - | - | 100 | - | - | 100 | 100 | - | 100 | - | 100 | - |
| Bendiocarb | 100 | 99 | 100 | 100 | 100 | 98 | 100 | 100 | 100 | - | 100 | - |
| Propoxur | - | 100 | 100 | - | - | 100 | 100 | - | 91 | - | 100 | - |

Data extracted from: *Report on Malaria vector susceptibility to public health insecticides in Uganda, September to October 2009*
