# Supplementary material for: Bioefficacy of long-lasting insecticidal nets against pyrethroid-resistant populations of Anopheles gambiae s.s. from different malaria transmission zones in Uganda
Source: Parasit Vectors. 2013 May 2;6:130. doi: 10.1186/1756-3305-6-130 (PMC3656772; doi:10.1186/1756-3305-6-130)
Supplement: Additional file 3 — Susceptibility to selected insecticides of adult An. gambiae s.l. from various districts in Uganda between October and November 2011. [file 1756-3305-6-130-S3.docx]

**Additional File 3**: 24-hour mortality (%) following exposure to selected insecticides in standard WHO susceptibility tests of adult *An. gambiae s.l*. from various districts in Uganda between October and November 2011. Test adults were non-blood fed and of known age (48-72 hours post-emergence), and were either the progeny of field-collected blood fed adult females or were reared from field-derived larvae.

| **Collection location** | **Wakiso** | | **Apac** | | **Kitgum** | | **Hoima** | | **Kanungu** | | **Tororo** | |
| --- | --- | --- | --- | --- | --- | --- | --- | --- | --- | --- | --- | --- |
| Origin of test adults | F1 of field adults | Field larvae | F1 of field adults | Field larvae | F1 of field adults | Field larvae | F1 of field adults | Field larvae | F1 of field adults | Field larvae | F1 of field adults | Field larvae |
| Vectors exposed (n) | 100 | 100 | 100 | 100 | 100 | 100 | 100 | 100 | 100 | 100 | 100 | 100 |
| DDT | 23 | - | - | 92 | - | 94 | - | - | - | 36 | 48 | - |
| Lambda-cyhalothrin | 30 | - | - | 36 | - | 65 | - | - | 53 | - | 34 | - |
| Etofenprox | - | 77 | - | 56 | - | 92 | 37 | 87 | 75 | - | 41 | - |
| Permethrin | - | - | - | 31 | - | - | - | - | - | - | 40 | - |
| Deltamethrin | 45 | - | - | 23 | - | - | - | - | 86 | - | 63 | - |
| Cyfluthrin | - | - | - | 40 | - | - | - | - | - | - | 27 | - |
| Alphacypermethrin | - | 98 | - | 69 | - | - | - | - | 100 | - | 89 | - |
| Pirimiphos-methyl | - | 100 | - | 100 | - | 100 | 100 | 100 | 100 | - | 100 | 100 |
| Malathion | - | - | - | 99 | - | - | - | - | - | - | 100 | - |
| Bendiocarb | 99 | - | - | 100 | - | 100 | 99 | 100 | 96 | 97 | 84 | 90 |
| Propoxur | - | - | - | 100 | - | - | - | - | - | 100 | 81 | - |

Data extracted from: *Report on Malaria vector susceptibility to public health insecticides in Uganda, October to November 2011*
